# Zinc oxide-ozonated olive oil as a new root canal filling material in primary molars: a clinical randomized controlled trial

**DOI:** 10.1007/s00784-023-05329-z

**Published:** 2023-10-21

**Authors:** Shaimaa S. EL-Desouky, Shaimaa M. Mahfouz Omer, Rehab F. Ghouraba, Reham M. Ali Abdel Latif, Ibrahim A. Kabbash, Shimaa M. Hadwa

**Affiliations:** 1 Pediatric Dentistry, Oral Health, and Preventive Dentistry Department, Faculty of Dentistry, Tanta University, Tanta, Egypt; 2 Pediatric Dentistry, Preventive Dentistry, and Dental Public Health Department, Faculty of Dentistry, Suez University, Suez, Egypt; 3 Oral Medicine, Periodontology, Oral Diagnosis, and Radiology Department, Faculty of Dentistry, Tanta University, Tanta, Egypt; 4 Pediatric Dentistry, Preventive Dentistry, and Dental Public Health Department, Faculty of Dentistry, Suez Canal University, Ismailia, Egypt; 5 Public Health & Community Medicine Department, Faculty of Medicine, Tanta University, Tanta, Egypt

**Keywords:** Pulpectomy, Primary molars, Ozonated olive oil, Zinc-oxide eugenol

## Abstract

**Objectives:**

The complex root canal anatomy of primary teeth keeps it very tricky to attain appropriate cleansing by biomechanical instrumentation, so obtaining an obturating material with excellent antimicrobial properties is a challenge in current clinical pulpectomy practice. So, this study aimed to assess the clinical and radiographic performance of zinc oxide-ozonated olive oil as a primary root canal filling material.

**Materials and methods:**

Ninety non-vital primary molars in children ranging from 4 to 8 years were allocated into three groups in which root canals were filled with zinc oxide-ozonated olive oil, zinc oxide-olive oil, and zinc oxide-eugenol (ZOE) according to each group after pulpectomy procedure. Clinical and radiographic evaluations were done at 3-, 6-, and 12-month follow-up periods. Statistical analysis was performed for the collected data.

**Results:**

All study groups showed a significant improvement regarding clinical signs and symptoms during follow-up periods. Ozonated-olive oil group revealed a significant increase in furcation radiodensity and a decrease in periodontal ligament space at 3-, 6-, and 12-month follow-up intervals compared to other groups.

**Conclusion:**

Zinc oxide-ozonated olive oil and zinc oxide-olive oil paste had shown good clinical and radiographic success for primary teeth pulpectomy.

**Clinical relevance:**

The intricate torturous primary root canal anatomy, in addition to the child’s negative behavior, interferes with the complete debridement, so the long-lasting antibacterial effect of the primary root canal filling material aids in the pulpectomy success.

## Introduction

Primary teeth pulpectomy entails root canal instrumentation followed by filling with a resorbable material [1]. Since 1930, ZOE has been the most prevalent filling material, with moderate to high success rates (more than 90%) [2, 3]. The main ZOE drawbacks are its limited antimicrobial activity [4] and the increased risk of erupting successor deviation in case of overfilled obturations [3]. Calcium hydroxide was also used alone or in combination with iodoform as a primary root canal filling material [5]. Despite their antibacterial and osteoinductive characteristics, calcium hydroxide and different iodoform pastes have the potential to fade from the canals before the primary root physiologic resorption [6].

Zinc eugenolate is formed when the crude form of eugenol (Clove oil) is mixed with zinc-oxide powder; its surface is easily hydrolyzed with free eugenol discharge which is detrimental to soft tissues [7]. At high concentrations, eugenol has a cytotoxic effect on fibroblasts and osteoblast-like cells [8], so it can induce necrosis and delayed healing [9]. Moreover, it acts as a contact allergen at lower concentrations, potentiating a localized delayed hypersensitivity reaction [10]. This inspired the quest for a novel alternative for eugenol to be combined with zinc oxide to overcome some of its disadvantages also, to develop chemical compounds with wider and more potent antibacterial activity.

Olive oil is a vegetal oil that contains 73% monounsaturated fatty acids and 55 to 83% oleic acid [11]. It also comprises antioxidants, oleuropein, carotenoids, and oleocanthal, a phenolic constituent that substantially promotes its antibacterial and anti-inflammatory characteristics [12, 13]. Ozone, a potent oxidizing agent, has been touted as promising owing to its biological properties, which include antibacterial activity, debriding effect, stimulation of angiogenesis, anti-inflammatory and analgesic effects, and immune response enhancement [5]. It is used in a variety of dental disciplines, including periodontology, surgery, pediatric dentistry, endodontics, and conservative dentistry [14]. Its applications involve periodontal pocket disinfection, caries prevention, healing acceleration, and tissue repair [15]. There are various forms of ozone, such as ozone gas, ozonated water, ozonated castor, and sesame oils, which are utilized for canal irrigation and as intracanal medicaments [16].

Ozonated olive oil, wherein the ozone particle is present as an ozonide among the double bonds of mono-saturated fatty acid, has the ability to produce nascent oxygen deeply through the treated area without triggering irritation [17]. Ozonated olive oil is obtained by ozone bubbling into the olive oil for an extended period of time. It is commonly used as a topical cream for skin conditions; it speeds up the growth of skin cells by enhancing wound healing and reducing inflammation [18]. It can also cure chronic periodontitis as an adjunctive therapy to scaling and root planning and it can be used as an intracanal medication [19]. Under the effect of ozone, better rheologic properties, raised intracellular ATP, enhanced cellular metabolism, and cytokine release linked to healing, mainly transforming growth factor (TGF-1), were noticed [20]. In order to find an ideal primary root canal filling material, this study aimed to assess and compare the clinical and radiographic performance of zinc oxide-ozonated olive oil and zinc oxide-olive oil as primary root canal filling materials compared to ZOE. The null hypothesis (H_0_) assumed that there was no difference in clinical and radiographic success after primary root canal filling using zinc oxide-ozonated olive oil, zinc oxide-olive oil, and ZOE at all follow-up periods.

## Materials and methods

### Study setting and ethical consideration

A prospective, controlled randomized clinical study was conducted at the Paediatric Dentistry Department Outpatient Clinic, Faculty of Dentistry, Tanta University, through the period from July 2021 to December 2022. The digital X-ray was done at the Department of Oral Medicine and Periodontology, Oral Diagnosis & Oral Radiology Department, Faculty of Dentistry, Tanta University. This trial was registered at ClinicalTrials.gov identifier NCT05633537. This study was approved by the ethical committee (REC), Faculty of Dentistry, Tanta University, code (#R-PED-1–21-2) complying with the Helsinki Declaration of 1964 and its subsequent amendments. Clinical treatment was started after parents signed written informed consent.

### Eligibility criteria

**Fig. 1:**
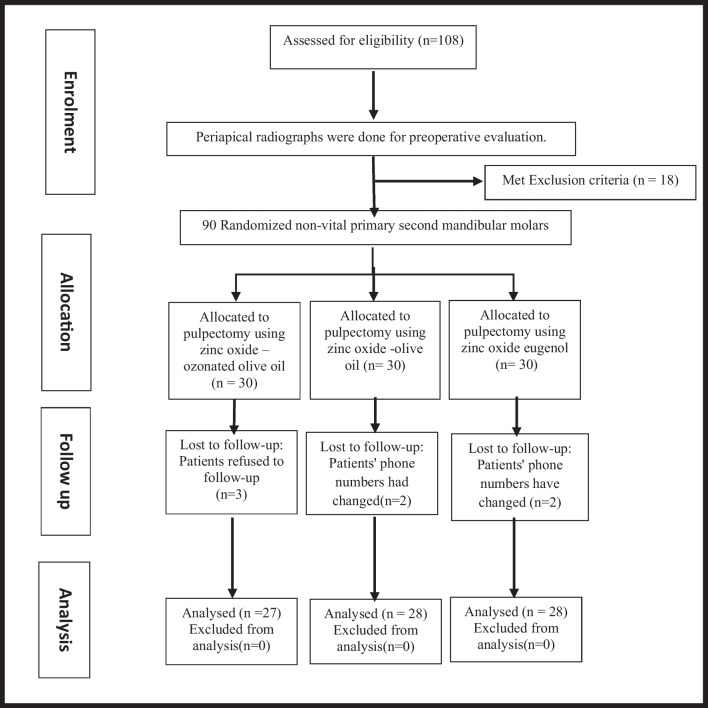
Flowchart explaining the child patient’s randomization and allocation throughout the clinical study

A total of 108 primary second molars in ninety-seven children with an age range of 4–8 years were enrolled and evaluated using the study's inclusion and exclusion criteria. Inclusion criteria were apparently healthy cooperative children having nonvital deep carious mandibular primary second molars with sufficient coronal structure and at least two-thirds of root structure intact. All selected children had a previous history of irreversible pulpitis signs and symptoms as spontaneous pain, percussion sensitivity, fistulous tract related to the involved tooth, pathologic tooth mobility (grade-I mobility), and the presence of interradicular radiolucency (discontinuity of lamina dura and/or furcation involvement less than or equal to half of the shortest root) [21]. Uncooperative children or those with systemic illness were excluded from this study. Also, non-restorable primary molars with severe mobility (greater than grade I mobility) [22] and/or extensive internal/external pathological root resorption were excluded. Accordingly, eighteen non-restorable primary molars were excluded so, the end study samples included 90 primary second molars. One molar per child was selected for this study. Figure 1 represents a flow chart that includes enrollment, allocation, assessment, and sample size analysis. All Clinical and radiographic signs and symptoms were recorded during patient examination before treatment.

### Sample size calculation and randomization

Using the Epi-Info statistical package developed by the World Health Organization and the Centres for Disease Control and Prevention, Atlanta, Georgia, USA, version 2002, the sample size and power analysis were calculated based on a previous study of Mortazavi and Mesbahi [23]. The criteria for sample size calculation were a 95% confidence limit, 80% power of the study, and expected successful clinical outcome at 60% among the least treatment group compared with 95% in the most favorable treatment groups. According to the previously specified criteria, the sample size in each group was 23; it was increased to 30 primary molars in each group to offset incomplete results and default rate. Simple randomization was computer-generated by the Research Randomizer software program (https://www.randomizer.org/) [24]. An independent person created a computer-generated randomized list that had been preserved in an ambiguous closed wrapper, to assign that met the inclusion requirements to any of the three groups.

### Group assignment

Ninety nonvital primary second molars were assigned into three groups (30 molar/group) at random according to different root canal filling materials:

- **Group-I (experimental group)** (*n* = 30): primary molars were filled with ozonated olive oil (Talya Bitkisel Ürünler, Türkiye) mixed with zinc oxide powder (PREVEST DenPro Limited, India).
- **Group-II (experimental group)** (*n* = 30): primary molars were filled with olive oil (Talya Bitkisel Ürünler, Türkiye) mixed with zinc oxide powder.
- **Group-III (positive control group)** (*n* = 30): primary molars were filled with ZOE (PREVEST DenPro Limited, India)**.**

### Pulpectomy procedure

A single visit pulpectomy procedure was carried out under strict aseptic conditions by a single trained operator (first author) who had experience in pediatric endodontic treatment. A perioperative evaluation was done using a digital intraoral photo-simulated phosphorus plate sensor (PSP, Planmeca ProSensor HD, Helsinki, Finland) before starting pulpectomy treatment. The teeth were anesthetized with 2% mepivacaine with 1:20,000 levonordefrin(Alexandria Co.) and isolated using a rubber dam (Midwest Dental, Texas, USA). Firstly, caries had been removed with sterile no. 330 carbide burs in high-speed contra-angle handpiece under copious water cooling and high suction. After gaining the access opening and removing the pulp chamber’s roof, an apex locator (J. Morita MFG Corp., Kyoto, Japan), was used to assess the working length, then affirmed by a periapical radiograph. The working length was determined as 1 mm less than the apex.

Root canals were explored for patency with #10 K-file also, to establish a glide path before instrumentation. Manual preparation with the # 20 K-file was done before introducing the rotary files. Then, all canals were instrumented using Kidzo rotary file system (Elephant dental supplies LTD, China) in a lateral brushing motion following the manufacturer’s instructions. The rotary files were used with an Endo-Mate DT endodontic motor (NSK, Tokyo, Japan) at 350 RPM and 2.5 N/cm torque in the following order: # 25/0.04 and # 30/0.04 [25]. Ethylenediaminetetraacetic acid (EDTA) gel (17%) (Meta Biomed Co. Ltd, Chungbuk, Korea) was applied to always lubricate the files during the biomechanical preparation. Irrigation was standardized to 5 ml of 1% sodium hypochlorite, followed by normal saline using a 23-gauge needle throughout the instrumentation procedure. In addition, a #10 K-file was applied to check canal patency after each file removal. Lastly, root canals were dried with sterile paper points # 30 (Dentsply Maillefer, OK, USA) before placement of the root canal filling materials using lentulo spirals according to each group.

Root canal filling was prepared as a fresh mix of 0.2 gm of zinc oxide powder mixed with 0.07 ml of ozonated olive oil, olive oil, and eugenol for groups I, II, and III respectively based on Tchaou et al.’s [26] formula. The precise amount of zinc oxide powder and liquid was measured using a digital weighing machine and a micropipette. Lastly, intermediate restorative material (reinforced zinc oxide eugenol, IRM® DENTSPLY International, USA) was placed, then a prefabricated stainless-steel crown (3 M™ ESPE, USA) was used as a final restoration. All groups were recalled at 3, 6, and 12 months for clinical and radiographic evaluations.

### Clinical and radiographic evaluation

The evaluation was carried out by two blinded trained pediatric dentists. At each follow-up period, the teeth were clinically assessed for the absence of swelling, tenderness with percussion, abnormal mobility, and spontaneous pain.

Radiographic assessment was done immediately following tooth restoration as a baseline and after that at 3, 6, and 12 months. Direct standardized digital radiographs were obtained with a photo-simulated phosphorus plate sensor (PSP, Planmeca ProSensor HD, Helsinki, Finland). The parallel periapical approach was achieved by the Rinn (XCP, Eign, IL) that was fitted to the x-ray tube with its arm fixed to the film holder which held a coated PSP (photo-stimulated phosphorous plate). Condensation rubber base impression material was placed on the outer surface of the film holder, and the child was instructed to bite on it during the material setting to guarantee standardization during radiographic image retakes. The sensor was subjected to an X-ray device (Planmeca ProX, Helsinki, Finland) at 70 kVp, 6 mA, and 0.8-s exposure time with perpendicular central ray to the sensor. Radio-densitometric and radiometric analyses of the radiographs were conducted using the histogram feature and length tool of Adobe Photoshop CS6 software (Adobe System, Inc., San Jose, CA). Radio-densitometric analysis was assessed by picking the regions of interest (ROIs) in middle of furcation area to quantity mean value of gray level while radiometric analysis was carried out by evaluating periodontal ligament widening through using measuring tool of software from base of furcation to deepest part of periodontal ligament space. Radiographic success was interpreted as a decrease or no change in preoperative pathologic inter-radicular radiolucency and a lack of new postoperative pathologic radiolucency also, decrease or no change in pre-operative periodontal ligament space.

### Statistical analysis

Data were gathered, tabulated, and statistically analyzed with SPSS version 19 (Statistical Package for Social Studies) developed by IBM, Illinois, Chicago, USA. The range, mean, and standard deviations of numerical variables were computed. The differences between the observations of bone density between groups were tested using a one-way analysis of variance. Meanwhile, the differences of bone density within each group were tested using repeated measurement analysis of variance. For other variables, which were not normally distributed, the differences of observations between groups were tested by Kruskal–Wallis test, and differences within groups at different follow-up periods were assessed by Friedman test. The significance level was set at *p* < 0.05.

## Results

All demographic data including age and gender distributions of enrolled children in each group were shown in Table 1. Kappa values for all assessments were greater than 0.85, demonstrating a high level of inter-examiner agreement. A total of ninety pulpectomy procedures were performed in mandibular primary molars in children ranging from4 to 8 years. All patients were presented for clinical and radiographic follow-up except three patients in group-I who refused to return in the follow-up periods, also two patients in group II & group III were lost because of changing their telephone number.

**Table 1:** Demographic distribution of study participants

Demographic characteristics	Group I (ozonated olive oil)		Group II (olive oil)		Group III (ZOE)	
	*n*	%	*N*	%	*n*	%
Age in years:						
4	9	30.0	10	33.3	11	36.7
5	8	26.7	8	26.7	10	33.3
6	8	26.7	5	16.7	3	10.0
7	5	16.7	6	20.0	6	20.0
8	0	0.0	1	3.3	0	0.0
Sex:						
Males	14	46.7	16	53.3	17	56.7
Females	16	53.3	14	46.7	13	43.3

### Clinical evaluation

All study groups showed a significant improvement regarding clinical signs and symptoms during follow-up periods. In the ozonated-olive oil group, at 3- and 6-month follow-up periods, all cases revealed no signs of spontaneous pain or gingival swelling while at twelve months, one case (3.7%) showed gingival swelling and spontaneous pain. Also, two cases (7.4%) sensed pain with percussion and exhibited mobility at a twelve-month follow-up period. Regarding the zinc oxide-olive oil group, two cases (7.1%) showed mobility at a 12-month follow-up period. On the other hand, mobility and pain with percussion were observed in five cases (17.9%) of the ZOE group at a twelve-month follow-up interval. Using the Friedman test, there were significant statistical differences in relation to all clinical variables within the same group at different follow-up periods (*P* < 0.05) as shown in Table 2. The most common failures at the 12-month follow-up period were tooth mobility, which represented 17.9%, 7.4%, and 7.1% of the children treated with ZOE, OZONATED OLIVE oil, and olive oil respectively. All failed cases were treated with extraction and space maintainer. The overall success rate was higher in groups I and II (92.6% and 92.9% respectively) than in group III (ZOE group) after a 12-month follow-up with no significant statistical difference (*p* = 0.346).

**Table 2:** The clinical evaluation findings among studied groups at different follow-up periods

Clinical criteria	Group I (ozonated olive oil) (*n* = 27)		Group II (olive oil) (*n* = 28)		Group III (ZOE) (i = 28)		Kruskal Wallis test	*p*
	*n*	%	*n*	%	*n*	%		
Swelling:								
Baseline	4	14.8	6	21.4	5	17.9	0.402	0.818
At 3 months	0	0.0	0	0.0	0	0.0	0.000	1.000
At 6 months	0	0.0	0	0.0	0	0.0	0.000	1.000
At 12 months	1	3.7	1	3.6	2	7.1	0.492	0.782
*Friedman test*	9.923		15.632		11.824			
*P*	0.019*		0.001*		0.008*			
Mobility:								
Baseline	5	18.5	6	21.4	6	21.4	0.094	0.954
At 3 months	2	7.4	2	7.1	1	3.6	0.445	0.800
At 6 months	1	3.7	1	3.6	1	3.6	0.001	1.000
At 12 months	2	7.4	2	7.1	5	17.9	2.125	0.346
*Friedman test*	7.714		8.429		13.105			
*p*	0.052		0.038*		0.004*			
Pain on percussion:								
Baseline	23	85.2	24	85.7	23	82.1	0.155	0.925
At 3 months	1	3.7	3	10.7	1	3.6	1.623	0.444
At 6 months	2	7.4	1	3.6	3	10.7	1.054	0.590
At 12 months	2	7.4	1	3.6	5	17.9	3.467	0.782
*Friedman test*	58.629		60.280		51.333			
*P*	< 0.001*		< 0.001*		< 0.001*			
Spontaneous pain:								
Baseline	21	77.8	21	75.0	21	75.0	0.076	0.963
At 3 months	0	0.0	0	0.0	0	0.0	0.000	1.000
At 6 months	0	0.0	0	0.0	0	0.0	0.000	1.000
At 12 months	1	3.7	1	3.6	2	7.1	0.492	0.782
*Friedman test*	60.188		60.188		57.738			
*P*	< 0.001*		< 0.001*		< 0.001*			
Overall success								
At 3 months	25	92.6	25	89.3	26	92.9	0.282	0.868
At 6 months	25	92.6	27	96.4	24	85.7	2.110	0.348
At 12 months	25	92.6	26	92.9	23	82.1	2.125	0.346
*Friedman test*	0.000		1.200		2.800			
*P*	1.000		0.549		0.247			

### Radiographic evaluation

**Fig. 2:**
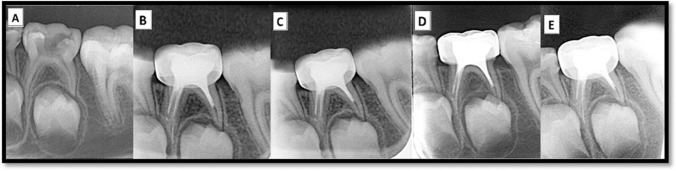
**A** Preoperative intraoral peri-apical (IOPA) radiograph of lower left second primary molar. **B** immediate IOPA radiograph post-obturation with zinc oxide-ozonated olive oil paste. **C-E** Post-IOPA radiographs at 3, 6, and 12 months

**Fig. 3:**
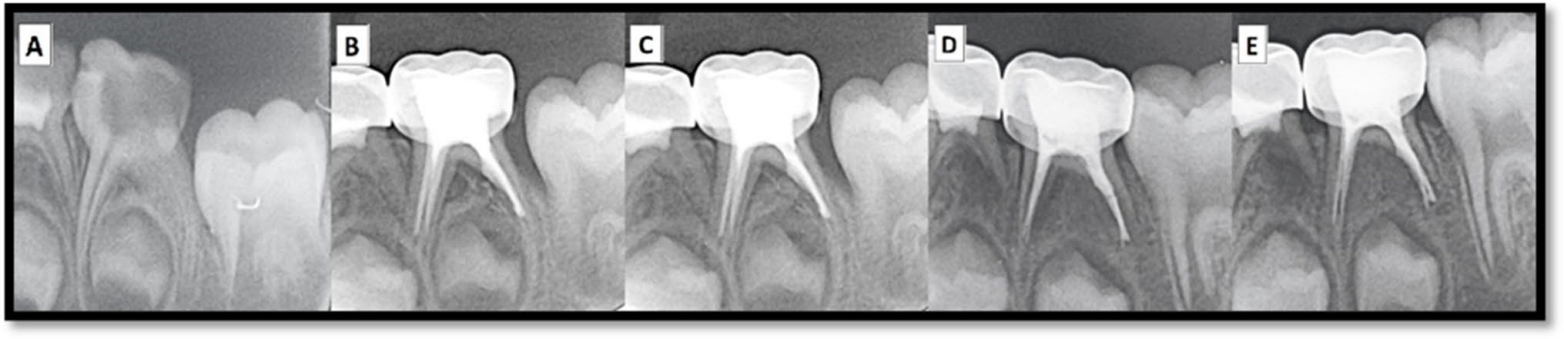
**A** Preoperative intraoral peri-apical(IOPA) radiograph of lower left second primary molar. **B** immediate IOPA radiograph post-obturation with zinc oxide-olive oil paste. **C–E** Post-IOPA radiographs at 3, 6, and 12 months

**Fig. 4:**
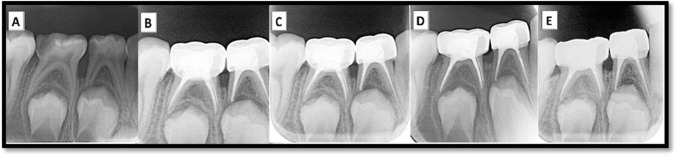
**A** Preoperative intraoral peri-apical (IOPA) radiograph of lower right second primary molars. **B** immediate IOPA radiograph post-obturation with ZOE paste. **C–E** Post-IOPA radiographs at 3, 6, and 12 months

Concerning furcation bone radiodensity, teeth filled with ozonated-olive oil revealed a significant increase in radiodensity at 3-, 6-, and 12-month follow-up periods, whereas ZOE-filled teeth presented a minor increase in bone radiodensity at all follow-up periods (Figs. 2, 3 and 4). In relation to the olive oil group, the radiographic evaluation revealed progressive healing of furcation radiolucencies over time (*p* < 0.001) (Fig. 3). There was a significant statistical difference at 3-, 6-, 12-month follow-up periods among the study groups (Table 3). By assessing the effect size between groups using pairwise comparisons, the ZOE group was significantly different from the other two groups at 3 and 6 months and it was only significantly different from the ozonated olive group at the 12-month follow-up period. Moreover, the effect size within each study group at different follow-up periods was statistically significant compared to the other two effect sizes. Regarding periodontal ligament (PDL) space, there was a significant decrease in PDL space at all follow-ups among the three groups (Figs. 2, 3, 4). By assessing the effect size within each group, each effect size was statistically significant compared to the other two effect sizes (*p* < 0.001). Kruskal Wallis test was done to compare between groups accordingly, there was no significant statistical difference at 3-, 6-, and 12-month follow-up periods as represented in Table 4.

**Table 3:** Comparison of bone density among studied groups at different follow-up periods

Bone density	Group I (ozonated olive oil)	Group II (olive oil)	Group III (ZOE)	*F*	*p*
Baseline:				6.852	0.002*
Range	34–46	35–55	33–55		
Mean + SD	39.00 ± 2.54	43.37 ± 6.60	41.50 ± 3.59		
At 3 months:				4.810	0.011*
Range	38–65	41–89	37–65		
Mean ± SD	56.96 ± 7.29	62.32 ± 11.09	53.32 ± 8.91		
Effect size:					
Range	− 2–26	− 5–48	− 5–24		
Mean ± SD	17.81 ± 6.65	18.68 ± 10.60	11.82 ± 9.20		
At 6 months:				3.591	0.032*
Range	35–93	32–98	33–93		
Mean ± SD	76.33 ± 15.18	79.43 ± 18.05	67.07 ± 18.98		
Effect size:					
Range	− 3–53	− 11–63	− 9–52		
Mean ± SD	37.18 ± 14.47	35.79 ± 18.21	25.57 ± 19.69		
At 12 months				6.249	0.003*
Range	32–135	32–135	32–132		
Mean ± SD	114.78 ± 29.05	106.64 ± 26.20	88.86 ± 31.33		
Effect size:					
Range	− 6–98	12–98	− 10–91		
Mean ± SD	75.63 ± 29.17	63.00 ± 27.88	47.36 ± 31.97		
*F* of effect size	83.563	53.887	35.250		
*p*	< 0.001*	< 0.001*	< 0.001*

**Table 4:** Comparison of periodontal ligament space widening among studied groups at different follow-up periods

Periodontal ligament widening	Group I (ozonated olive oil)	Group II (olive oil)	Group III (ZOE)	Kruskal Wallis test	*p*
Baseline:				6.561	0.038*
Range	0.17–0.23	0.17–0.24	0.19–0.23		
Mean ± SD	0.20 ± 0.02	0.21 ± 0.02	0.20 ± 0.02		
At 3 months:				0.528	0.768
Range	0.16–0.21	0.17–0.23	0.17–0.23		
Mean ± SD	0.19 ± 0.01	0.19 ± 0.02	0.19 ± 0.02		
Effect size:					
Range	− 0.03–0.02	− 0.03–0.00	− 0.03–0.03		
Mean ± SD	− 0.01 ± 0.01	− 0.01 ± 0.01	− 0.01 ± 0.02		
At 6 months:				3.644	0.162
Range	0.15–0.21	0.16–0.23	0.16–0.24		
Mean ± SD	0.17 ± 0.02	0.18 ± 0.02	0.19 ± 0.02		
Effect size:					
Range	− 0.06–0.01	0.05–0.02	− 0.06–0.04		
Mean ± SD	− 0.03 ± 0.02	− 0.02 ± 0.02	− 0.02 ± 0.03		
At 12 months				3.510	0.173
Range	0.15–0.22	0.15–0.25	0.15–0.25		
Mean ± SD	0.17 ± 0.02	0.17 ± 0.02	0.18 ± 0.03		
Effect size:					
Range	− 0.07–0.04	− 0.05–0.06	− 0.07–0.05		
Mean ± SD	− 0.03 ± 0.03	− 0.03 ± 0.02	− 0.02 ± 0.03		
Friedman test	51.660	60.781	22.008		
*P*	< 0.001*	< 0.001*	< 0.001*		

## Discussion

Primary tooth preservation in a functional condition until its normal shedding is highly important in pediatric dentistry [27]. It is incredibly challenging to carry out a perfect pulpectomy procedure due to the presence of complicated, tortuous root canals as well as the risk of permanent tooth injury [28]. An ideal pulpectomy material should be safe for the developing permanent teeth and periapical tissues, resorbable, and have antibacterial effects [29]. The purpose of this study was to evaluate the clinical and radiographic performance of three root canal filling materials in primary teeth pulpectomy to find an ideal or near-ideal obturating material.

In this study, other vehicles were mixed with zinc oxide powder as ozonated olive oil which was chosen because ozone is unsteady in a gaseous state and needs special equipment to be present chairside, and when ozone liquifies in water, it quickly breaks down, making it impossible to store [30]. Children aged 4 to 8 years were chosen for this study, regardless of their sex; this is consistent with Doneria et al. [31] and Tirupathi et al. [32]. This age range was chosen to evade the younger children’s lack of cooperation and the physiological root resorption in the elder children.

The present study results accepted the null hypothesis as there was no statistically significant difference regards the overall clinical success of the three groups at all follow-up periods, whereas a significant difference was observed regarding furcal bone radio-density among the study groups at all follow-up periods (null hypothesis was partially rejected). The overall clinical evaluation of ozonated olive oil group at all follow-up periods revealed excellent clinical signs of success; this promising clinical outcome could be explained by ozone peroxides’ potent antibacterial effects and excellent healing characteristics. The ozonated olive oil’s antimicrobial activity may be due to the effects of aldehydes, unsaturated fatty acids, and hydrogen peroxide on anaerobic bacteria frequently detected in endodontic infection [19]. This is in agreement with Doneria et al. [31] who compared zinc oxide-ozonated castor oil, 3-Mix-antibiotic paste, and Vitapex in primary molar pulpectomy and found 100% clinical success rate at 6 and 12 months. Also, these findings coincided with Chandra et al. [5] who assessed ZO-ozonated sesame oil, Vitapex, and 3-Mix antibiotic paste as primary root canal filling materials and found a 100% success rate at a 12-month follow-up period. Moreover, this is consistent with Wassef and Fouad, [16] who reported a greater antibacterial effect of ozonated olive oil contrasted to sodium-hypochlorite with increased inhibition zones against *Enterococcus faecalis* bacteria. On the other hand, this was in contrast with Kalaskar et al.’s [33] study which concluded that the calcium hydroxide group showed a significant reduction in bacterial colony counts than ozonated olive oil group. Also, this study results disagreed with Elshinawy et al. [34] who reported that chitosan nanoparticles outperformed silver nanoparticles and ozonated olive oil in terms of antimicrobial activity against the assessed endodontic bacteria. In addition, Mittal et al., [35] observed that NaOCl showed the highest bacterial count reduction, followed by ozonated olive oil; the least bacterial reduction of Ozonated olive oil may be explained by the ozonolysis reaction is meaningless if peroxide released by ozonated oil could not be quantified [36]. Moreover, a study conducted by Padmakumar et al. [37] had found that NaOCl substantially decreased the dentin’s organic components like carbonate and phosphate in comparison to ozonated olive oil and silver citrate as new irrigating solutions; this may be linked to the viscous nature of ozonated olive oil which restricts its penetration.

Regarding the olive oil group, the overall clinical success at all follow-up times revealed comparable results to ozonated olive oil group; this agreed with Feier et al. [38] who evaluated the efficacy of pure olive oil and ozonated olive oil in gingivitis treatment and found that both tested groups had anti-inflammatory and antimicrobial effects. This may be attributed to its high antimicrobial effects as the unsaturated fatty acids in olive oil can incorporate into the cytoplasmic membrane of the bacteria, causing lethal structural disturbances and disrupting the membrane integrity [19]. Moreover, this is agreed with El-Shafy et al. [12] who evaluated the antimicrobial effect of olive oil extract compared to chlorhexidine as mouthwashes in children and reported a high antimicrobial activity of olive oil towards Gram-positive and Gram-negative bacteria.

In this study, the overall ZOE group evaluation reported an acceptable clinical success rate at all follow-up periods; this may be explained by the anti-inflammatory and analgesic properties of eugenol since the released-eugenol amount instantly after application in the periapical zone is 10^−4^ and drops to 10^−6^ after 24 h then reaches zero after one month. At 12 months follow-up, the clinical success rate of the ZOE group was 82.1% which was lower than the results of Trairatvorakul and Chunlasikaiwan [21] study that reported 85% clinical ZOE success rate versus 89% success rate in calcium hydroxide-iodoform paste group in pulpectomized primary molars. On the other hand, this study’s results were relatively greater than Mortazavi and Mesbahi’s [23] study which showed a 78.5% success rate in the ZOE group compared to a 100% success rate in Vitapex group. This results conflict might be attributed to the differences in pulpectomy procedure including mechanical preparation as well as root canal irrigants.

No significant statistical difference among the three groups was detected at 3, 6, and 12 months with a satisfactory clinical success rate; this excellent overall outcome might be attributed to careful case choice, appropriate protocols, and proper material usage. Additionally, the stainless-steel crowns offer the best coronal seals against microbial leakage in primary molars. Ozonated olive oil group & olive oil group had a considerable clinical success rate than the ZOE group with no statistically significant difference; this agreed with Nardi et al. [39] who observed a great bacterial inhibition zone of ozonated oil and pure olive oil against *Streptococcus mutans*. In the current study, the most dominantly observed failures at the 12-month follow-up period were abnormal tooth mobility; this concurred with Trairatvorakul and Chunlasikaiwan’s [21] study that stated 11% of cases with pathological tooth mobility.

Regarding furcal bone radio-density, a statistically significant difference was observed at the 3, 6, and 12-month follow-up intervals among the study groups with a progressive furcal bone regeneration in the ozonated olive oil group; this agreed with Feier et al. [38]. These results may be linked to the chemical reaction of ozone with carbon–carbon double bonds in the unsaturated fatty acids which results in lengthy complicated molecules known as “ozonoids” that encourage the repairing and regenerative mechanisms [17]. In relation to the periodontal ligament (PDL) space, there was a significant reduction in PDL space at all follow-up periods among the three groups with a significant decrease in the ozonated olive oil group; this was in line with El-Shafy et al. [12] and Nardi et al. [39]. This could be explained by ozone stimulation of growth factors that are essential for controlling inflammatory responses as well as promoting wound healing.

To the best of our knowledge, this is the first study assessing ZO-ozonated olive oil paste compared to olive oil and ZOE as primary root canal filling materials. According to the results of this study, ZO-ozonated olive oil paste can be a substitute for ZOE, producing acceptable clinical and radiographic outcomes. Even so, more clinical, and histologic research with a longer follow-up period is required to reach definitive conclusions. The present research has limitations, including a small sample size and short follow-up periods while its results can be used as a basis for future studies. Moreover, the effect of tested materials on exfoliation time was not measured which can be done in upcoming randomized clinical trials with long-term evaluation periods before a reliable conclusion can be drawn as the best root canal filling material for primary teeth. Also, the concentration of ozone in ozonated olive oil was not given in this study which may produce unexpected oxidative reactions at different concentrations of ozone, so further experiments are necessary to investigate this effect.

## Conclusion

The study findings suggest that primary root canal filling using zinc oxide powder combined with ozonated olive oil also, with olive oil has shown good clinical and radiographic success and can be regarded as an alternative filling material for pulpectomy treatment of infected primary teeth.

## Data Availability

On reasonable request, the datasets utilized and/or analyzed during the present study are accessible from the corresponding author.
